# Hsa-mir-1293/GLI1/PTCH1 axis is involved in proliferation, migration, and EMT of laryngeal cancer

**DOI:** 10.1016/j.bjorl.2026.101860

**Published:** 2026-07-22

**Authors:** Yibo Zhou, Yu Ren, Zhi Chen, Jun Wan, Yongchuan She, Yi Tang, Dan Yang, Hong Guo, Zhongxi Huang

**Affiliations:** a Changsha Hospital of Traditional Chinese Medicine (Changsha Eighth Hospital), Department of Otolaryngology Head and Neck Surgery, Changsha, China; b The First Affiliated Hospital of Guangxi Medical University, Department of Otolaryngology Head and Neck Surgery, Nanning, China; c Binhai New Area Hospital of Traditional Chinese Medicine, The Fourth Affiliated Hospital of Tianjin University of Traditional Chinese Medicine, Tianjin, China; d Southern Medical University, School of Basic Medical Sciences, Cancer Research Institute, Guangzhou, China

**Keywords:** Laryngeal cancer, Epithelial-mesenchymal transition, GLI1, PTCH1, hsa-mir-1293

## Abstract

**Objective:**

This study aimed to investigate the specific role and molecular mechanisms of the transcription factor GLI1 and its upstream regulatory miRNA, hsa-mir-1293, in laryngeal cancer development, with a focus on elucidating the function of the hsa-mir-1293/GLI1/PTCH1 signaling axis in regulating laryngeal cancer cell proliferation, migration, and EMT.

**Methods:**

Differentially expressed miRNAs were screened from TCGA laryngeal cancer miRNA data, and the interaction between hsa-mir-1293 and GLI1 was validated using a dual luciferase reporter assay. The effects of hsa-mir-1293 on TU212 cell proliferation, clonal formation, and invasion were examined. The role of GLI1’s downstream target, PTCH1, in cell proliferation, migration, and EMT was further investigated.

**Results:**

Bioinformatics analysis identified hsa-mir-1293 as the most significantly down-regulated miRNA in laryngeal carcinoma, showing a negative correlation with GLI1 expression. Overexpression of hsa-mir-1293 suppressed TU212 cell proliferation, clonal formation, and invasion. Silencing GLI1 reduced PTCH1 expression, while PTCH1 overexpression promoted cell invasion, migration, and EMT.

**Conclusion:**

Hsa-mir-1293 targets GLI1, and the GLI1/PTCH1 axis plays a critical role in laryngeal cancer cell proliferation, migration, and EMT.

**Level of evidence:**

5.

## Highlights

• The hsa-mir-1293/GLI1/PTCH1 axis is a novel pathway in laryngeal cancer.
• Hsa-mir-1293 is down-regulated and directly targets GLI1.
• Hsa-mir-1293 suppresses tumor cell proliferation and migration.
• GLI1 promotes cancer via transcriptional activation of PTCH1.
• PTCH1 enhances tumor invasion and epithelial-mesenchymal transition.

## Introduction

Laryngeal cancer, mostly squamous cell carcinoma, is a common malignant tumor of the head and neck. Early-stage patients can be treated with surgery or radiotherapy, while advanced cases require multimodal therapy. Although treatments have improved over the past 40-years, the 5‑year survival rate has not increased significantly. Invasion and metastasis are major causes of poor prognosis, in which Epithelial‑Mesenchymal Transition (EMT) acts as a key driver.^1^^,^^2^

The Hedgehog (Hh) signaling pathway is critical for embryonic development and tissue homeostasis, and its abnormal activation contributes to tumor progression and EMT.^3^ Using bioinformatics analysis, we divided laryngeal cancer into two molecular subtypes (C1 and C2). The more aggressive C2 subtype showed significant enrichment of the Hh pathway and high expression of its downstream transcription factor GLI1.^4^ Originally identified as an oncogene in glioma, GLI1 upregulation serves as a hallmark of Hedgehog (Hh) signaling pathway activation, has been widely detected in multiple tumor types,^5^ and is closely associated with malignant processes including tumor invasion, metastasis, differentiation, prognosis, and EMT.^6^, ^7^, ^8^, ^9^, ^10^, ^11^, ^12^, ^13^, ^14^, ^15^, ^16^, ^17^

PTCH1 (Patched 1), a key membrane receptor and negative feedback regulator of the Hh pathway, relieves Smoothened (SMO) inhibition via binding Hh ligands.^18^^,^^19^ As a classic downstream target of GLI1, PTCH1 is transcriptionally activated by GLI1 through direct promoter binding, forming a GLI1-PTCH1 negative feedback loop to maintain Hh pathway homeostasis.^20^ Dysregulated PTCH1 expression induces sustained Hh activation and promotes tumorigenesis in in multiple malignancies,^21^, ^22^, ^23^, ^24^ but its regulatory relationship with GLI1 and their combined role in laryngeal cancer EMT remain unclear.

Although the overexpression of GLI1 is significant in laryngeal cancer, its upstream regulatory mechanisms and downstream target genes remain unclear. MicroRNAs (miRNAs) are small non-coding RNAs that regulate gene expression post-transcriptionally and participate in multiple biological processes.^25^ We hypothesized that miRNAs may be important upstream regulators of GLI1. In this study, we used TCGA data and bioinformatics analysis to screen candidate miRNAs targeting GLI1. We further verified their effects on proliferation, migration, and EMT of laryngeal cancer cells in vitro. We also explored key downstream targets of GLI1 to clarify its molecular mechanism in promoting EMT, providing an experimental basis for studying GLI1-mediated chemoresistance and radioresistance in laryngeal cancer.

## Methods

### Cell culture and treatment

Human laryngeal cancer cells TU212 were purchased from Department of Otolaryngology-Head and Neck Surgery School of Medicine, Changsha University (Changsha, China), and human pharyngeal epithelial cells NP69 were kindly donated by Institute of Oncology, The First Affiliated Hospital of Guangxi Medical University (Nanning, China). The cell lines were authenticated by the original providers via Short Tandem Repeat (STR) profiling prior to our acquisition, and all experiments were performed using low-passage cultures (≤ 15 passages) to maintain the biological characteristics and avoid cross-contamination. Expression of GL1 gene stable strain and interference with GL1 gene stable strain were purchased from IEMed Guangzhou Biomedical Technology Co., Ltd. TU212 and NP69 cells were cultured in RPMI1640 complete culture medium containing 10% FBS (Gibco, USA) + 1% double antibody (Solarbio, Beijing, China) (Gibco, USA), placed in a cell culture incubator (37 °C, 5% CO_2_), every 1–2 days to observe the growth morphology of the cells, the condition of the culture medium, half or full replacement of the culture medium. When the cell culture coverage reached 80%∼90%, the superannuate was discarded, washed 2–3 times with PBS (Gibco, USA), digested with 0.25% trypsin (Gibco, USA) for 2–4 minutes, observed by inverted microscope, and then transferred to a sterile centrifuge tube for 4-minutes, and then diluted with 5 mL of medium and transferred to another sterile culture flask for further culture.

### Data set

Transcript expression profiles of Head and Neck Squamous Cell Carcinoma (HNSCC) patients and their clinical data (n = 565) were obtained from the UCSC Xena database (https://tcga.xenahubs.net/download/TCGA.HNSC.sampleMap/HiSeqV2.gz, Full metadata). Among the 565 HNSCC patients, 126 had an “anatomic_neoplasm_subdivision” (tumor anatomical site) of “Larynx” (larynx), which included 114 primary tumors and 12 neoplastic tissues. Gene expression profiling of 109 primary laryngeal cancer samples was downloaded from Gene Expression Omnibus (GEO, GSE27020). To identify possible target genes of GLI1, ChIP-seq data of GLI1 was obtained from GEO (GSE42132). To construct a conscription regulatory network, a list of 1469 genes encoding transcription factors was downloaded from (http://bioinfo.life.hust.edu.cn/static/AnimalTFDB3/download/Homo_sapiens_TF).

### GLI1 upstream miRNA prediction and screening

Using the SAM method with R.fold > 1.5 and a mean expression value of regulated subgroups > 3, screen for C2 expression-regulated and downregulated miRNAs in TCGA's miRNA data (TCGA_HNSC_miRNA_HiSeq-2015-02-24). Querying the database of miRNA target genes mirDIP (http:// ophid.utoronto.ca/mirDIP/index.jsp) to screen the genes regulated by candidate miRNAs.

### Plasmid construct and transfection

For GLI1 expression, full-length GLI1 cDNA was amplified and cloned into the vector pCDH-CMV-MCS-EF1-copGFP-T2A-Puro. Expression mimic and interference inhibitor were designed and prepared against hsa-mir-1293, using hsa-mir-1293 mimic instead of mir-1293, and inhibitor to inhibit the expression of mir-1293, using mimic nc and inhibitor nc as control. For transfection, cells were cultured in 6-well plates until 70% confluence, plaids were transacted using trifecta CP Transfection kit (RuiBo Bio, Guangzhou, China), and hsa-mir-1293 mimic/inhibitor was added to the cells and incubated for 48 h after transfection.

### Real-time quantitative reverse transcription PCR (qRT-PCR)

Total RNA was extracted from the nontransferable cells using Trizol reagent, cDNA was synthesized by reverse transcription, and the expression levels of mRNA and miRNA were quantified using SYBER Premix Ex TaqTM. GAPDH or U6 was used as the internal reference gene.

### Wound healing assay

Cells were inoculated and cultured in 12-well plates, and after transfection for 8 h, the original medium was discarded and replaced with complete medium to continue culturing for 16 h. Cells were scratched with the tip of a plastic 10 μL pipette gun tip, and washed with PBS for 3-times. Fresh growth medium without FBS was added to the plates. Subsequently, they were placed in a 37 °C 5% CO_2_ incubator for incubation. Images of the different stages of wound healing were taken at 0 and 24 h by microscope.

### Transwell migration and invasion

Cells were inoculated in Transwell plates with serum-free medium, and after transfection for 8 h, the original medium was discarded and replaced with complete medium to continue incubation for 16 h, and cell status and density were observed. Add 10% FBS medium into the lower chamber of Transwell. At the end of culture, the upper chamber was removed and put into a new 24-well plate, and PBS was washed gently for 3-times. Add 4% paraformaldehyde into the small chamber and fix it at room temperature for 15 min, add crystal violet staining solution to stain the cells, wash away the excess staining solution with PBS, remove the upper layer of cells from the upper chamber with a cotton swab, then dry it at room temperature and observe it under the microscope.

### Western blot analysis

For protein blot analysis, total protein samples were extracted from transfected shGLI1−1, shGLI1−2, shGLI1−3, TU212-shPTCH1, TU212-PTCH1 cells using RIPA lysate. Equal amounts of proteins were up sampled onto 8% SDS-polyacrylamide gels and then transferred to PVDF membranes. The membranes were next closed in in 5% BSA solution for 1 h and then incubated with primary antibodies against GLI1 (Abcam, Cat# ab134906), GLUL (Abcam, Cat# ab176562), MAP1B (Abcam, Cat# ab154333), PTCH1 (Abcam, Cat# ab53715), GAPDH, E-cadherin (Abcam, Cat# ab40772), N-cadherin (Abcam, Cat# ab245117), Snail (Abcam, Cat# ab216347), Vimentin (Abcam, Cat# ab92547) primary antibodies at 4 °C overnight. The membrane was then incubated with secondary antibodies Hrp-Goat Anti-Rabbit IgG (Jackson, Cat# 111-035-003), Hrp-Goat Anti-Mouse IgG (Jackson, Cat# 115-035-003). Finally, immunoreactivity was detected using a chemiluminescent imaging system (Tanon, China).

### GLI1 dual luciferase reporter gene plasmid construction

Cells were inoculated into 6-well plates and transacted for 8 h. After transfection, complete medium was replaced, and incubation continued for 40 h. The medium was discarded, and the cells were dialyses. Firefly luciferase substrate (50×) and sea kidney luciferase substrate (50×) were diluted to 1× working solution with corresponding buffers. Firefly luciferase reaction solution and sea kidney luciferase reaction solution were added to the stately to detect the luciferase activity.

### Statistical analysis

Statistical analysis was performed using SPSS 19.0 software. All data were presented as mean ± standard deviation from at least three independent biological replicates. Comparisons between two groups were conducted using Student's *t*-test. For experiments involving more than two groups, one-way ANOVA followed by Dunnett’s post-hoc test was applied for multiple comparisons. Differences were considered statistically significant when p < 0.05.

## Results

### miRNAs screening and prediction results

In the TCGA miRNA data (TCGA_HNSC_miRNA_HiSeq-2015-02-24), consistent with the C1 and C2 subtypes, there were 103 cases, split between 60 cases and 43 cases. Using the SAM method, with R.fold > 1.5 and the mean of the expression values of the upregulated miRNAs > 3, a total of 2 miRNAs were identified as upregulated, while a total of 10 miRNAs were identified as downregulated for the C2 subtype (Table 1). As shown in Table 1, the down-regulated miRNAs in C2 are most significantly down-regulated in hsa-miR-1293, with C2 showing only 0.36-fold of C1 and a q-value of 0.

**Table 1:** Differentially expressed miRNAs of different molecular subtypes of TCGA.

miRNA	d-value	stdev	p-value	q-value	R.fold	Mean_C1	Mean_C2
hsa-mir-204	4.78	0.39	1.20E-06	4.52E-05	3.58	1.36	3.20
hsa-mir-769	4.90	0.14	1.20E-06	4.52E-05	1.59	4.58	5.25
**hsa-mir-1293**	**−6.11**	**0.24**	**0.00E+00**	**0.00E+00**	**0.36**	**3.53**	**2.06**
hsa-mir-193b	−6.50	0.19	0.00E+00	0.00E+00	0.42	7.45	6.21
hsa-mir-205	−4.57	0.19	3.59E-06	1.23E-04	0.55	14.00	13.13
hsa-mir-2355	−4.50	0.17	7.19E-06	2.26E-04	0.59	6.45	5.68
hsa-mir-31	−4.89	0.38	1.20E-06	4.52E-05	0.28	6.37	4.52
hsa-mir-338	−4.33	0.20	1.56E-05	4.52E-04	0.56	8.44	7.59
hsa-mir-365-1	−6.03	0.16	0.00E+00	0.00E+00	0.52	5.18	4.23
hsa-mir-365-2	−6.15	0.15	0.00E+00	0.00E+00	0.53	5.10	4.19
hsa-mir-455	−4.74	0.22	1.20E-06	4.52E-05	0.48	8.68	7.63
hsa-mir-576	−4.74	0.13	1.20E-06	4.52E-05	0.65	3.71	3.08

Mean_C1 and Mean_C2 represent the log2 mean expression values of C1 and C2 subtypes, respectively.

In the previous study,^1^ in order to identify the possible target genes of GLI1, ChIP-seq data for GLI1 was obtained from GEO (GSE42132). Since GLI1 is a transcription factor, three up-regulated genes (PTCH1, GLUL, and MAP1B) associated with laryngeal cancer C2 subtype were screened for their tendency to be recognized and bound by GLI1 based on the ChIP-seq data (GSE42132).

**Fig. 1:**
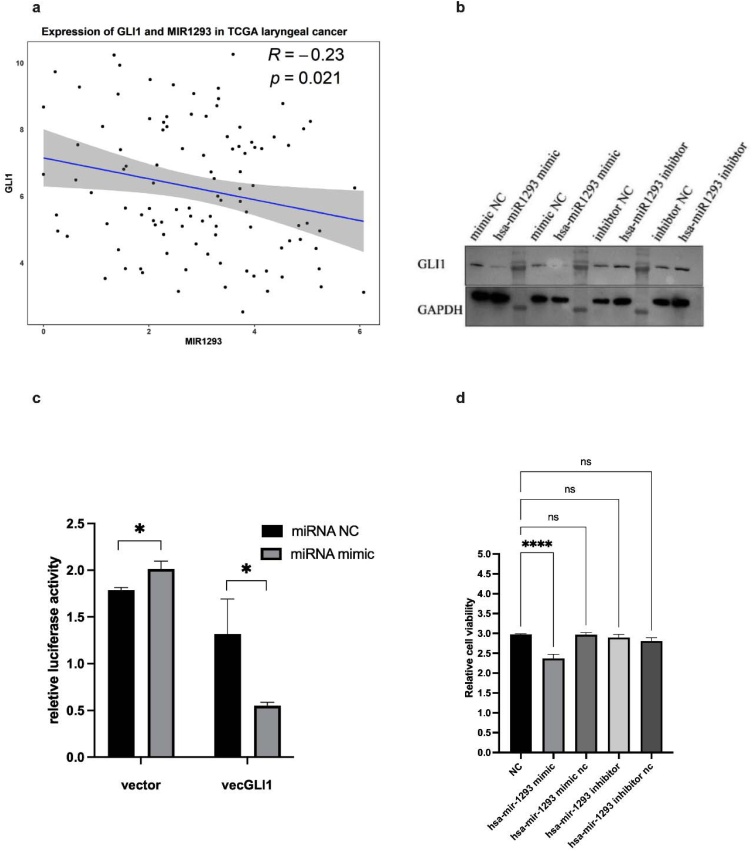
Hsa-miR-1293 targets GLI1 and regulates cell cycle and proliferation in TU212 cells. (a) Scatter plot of hsa-miR-1293 versus GLI1 expression (log2) in TCGA laryngeal cancer data shows a significant negative correlation (Pearson *r* = −0.23, p = 0.021). (b) Western blot confirms hsa-miR-1293 negatively regulates GLI1 expression (*p < 0.05). (c) Dual luciferase assay demonstrates hsa-miR-1293 directly targets GLI1, reducing luciferase activity (* p < 0.05). (d) CCK8 assay shows hsa-miR-1293 overexpression reduces cell viability (**** p < 0.0001). ns, not significant.

Querying the miRNA target gene database mirDIP, hsa-mir-1293 was found to co-regulate GLI1 and its downstream signaling molecules GLI2 and PTCH1 (Table 2). In the data of 103 laryngeal cancers with both mRNA and miRNA detected in TCGA, the expression (log2) scatter plot of has-mir-1293 versus GLI1 was plotted (Fig. 1a). The results revealed that has-mir-1293 was significantly negatively correlated with GLI1 expression (Pearson correlation coefficient *r* = −0.23, p = 0.021).

**Table 2:** Hsa-mir-1293 co regulates GLI1 and its downstream molecules.

Gene	MicroRNA	Score	N	Class	Sources
GLI1	hsa-miR-1293	0.02	2	Medium	BCmicrO|RNAhybrid
GLI2	hsa-miR-1293	0.24	9	High	BCmicrO|BiTargeting|DIANA|ElMMo3|MBS
PTCH1	hsa-mir-1293	0.15	8	Medium	BCmicrO|BiTargeting|DIANA|MBStar|MirAncesTar|MirMAP|RepTar|RNAhybrid

### hsa-mir-1293 can target GLI1 gene

**Fig. 2:**
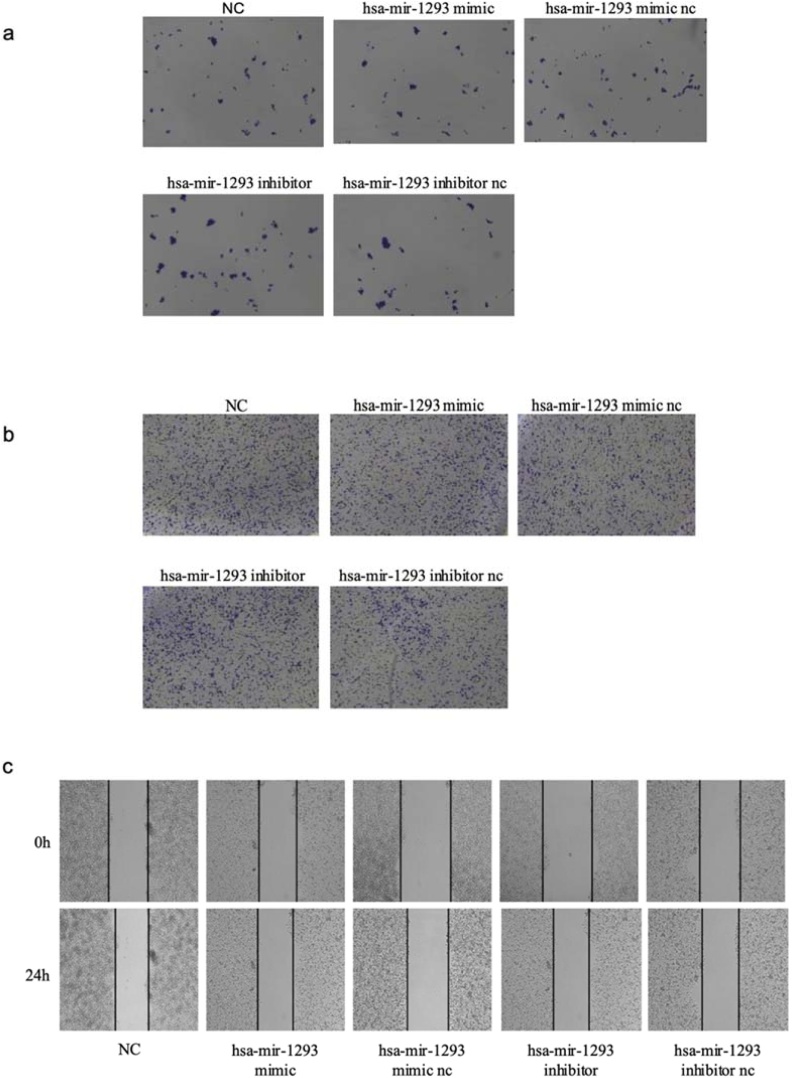
Hsa-miR-1293 regulates clone formation, invasion, and migration in TU212 cells. (a) Clone formation assay shows hsa-miR-1293 overexpression reduces cell colony formation (NC; Scale bar = 200 μm). (b) Transwell assay reveals hsa-miR-1293 inhibition increases cell invasion ability, while mimic transfection has no significant effect (NC; Scale bar = 500 μm). (c) Cell scratch assay demonstrates hsa-miR-1293 overexpression decreases cell migration ability (NC; Scale bar = 200 μm).

In the TCGA laryngeal cancer mRNA and miRNA data, hsa-miR-1293 was significantly negatively correlated with GLI1 expression, suggesting that hsa-miR-1293 is a candidate upstream miRNA for GLI1. We transfected TU212 cells with hsa-mir-1293 mimic or inhibitor. RT-qPCR (Fig. S1C) and western blot assays showed that the expression of hsa-miR-1293 was negatively related to the expression of GLI1 (p < 0.05; Fig. 1b). To investigate the effect of hsa-miR-1293 on GLI1, we transfected TU212 cells with a dual luciferase reporter gene plasmid containing hsa-miR-1293 and GLI1. The luciferase activity of the transfected hsa-miR-1293 mimic GLI1 reporter gene vector was significantly reduced compared to the control vector, indicating that hsa-miR-1293 could target the GLI1 gene (Fig. 1c). The results of the cell cycle assay showed that in TU212 cells overexpressing hsa-miR-1293, the proportion of cells in the G1 phase was higher than that in the control group. Conversely, when hsa-miR-1293 was interfered with, the proportion of cells in the G1 phase was lower than that in the control group (p < 0.05, Fig. S2A). To investigate the biological function of hsa-mir-1293, CCK8 cell proliferation assay showed that overexpression of hsa-mir-1293 in TU212 cells decreased cell viability (p < 0.0001) (Fig. 1d). Clone formation assay showed that overexpression of hsa-mir-1293 resulted in diminished cell clone formation (p < 0.05, Fig. 2a). In addition, Transwell assay showed that hsa-mir-1293 mimic transfection of TU212 cells resulted in no significant change in cell invasion ability, but inhibition of hsa-mir-1293 expression increased cell invasion ability (p < 0.05, Fig. 2b). Cell scratch assay showed that overexpression of hsa-mir-1293 decreased cell migration ability (p < 0.05, Fig. 2c).

### GLI1-dependent regulation of PTCH1

**Fig. 3:**
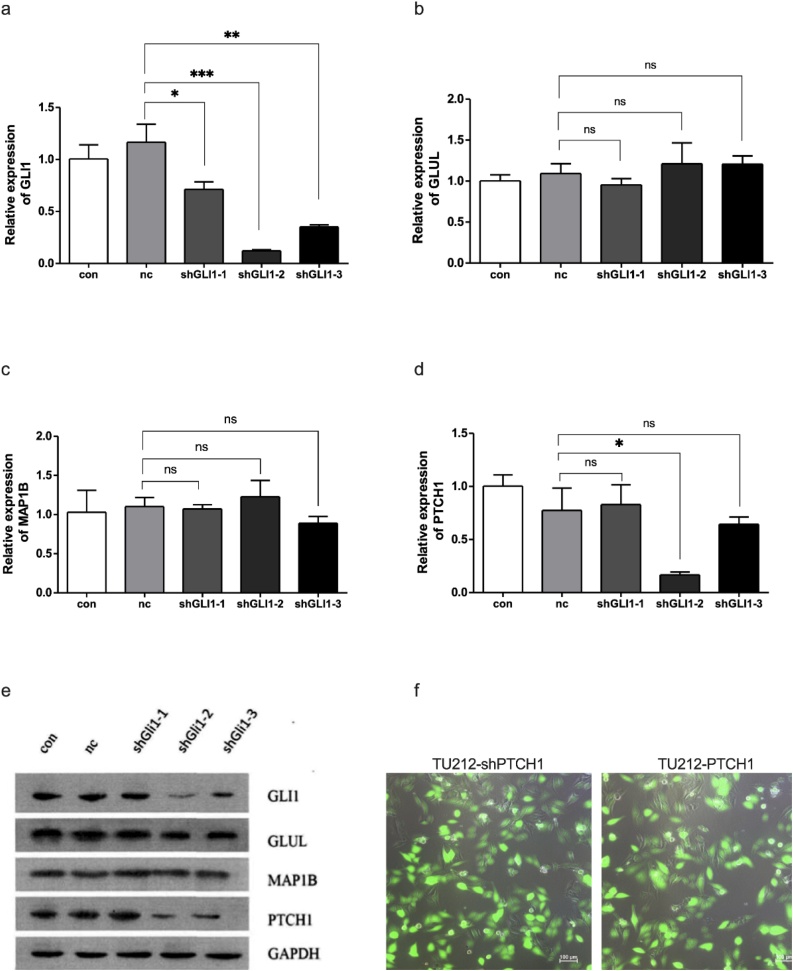
Silencing GLI1 regulates PTCH1 expression. (a) TU212 cells were transfected with siRNAs of different sequences, and the mRNA expression levels of GLI1, * p < 0.05, ** p < 0.01, *** p < 0.001. (b) GLUL, ns, not significant. (c) MAP1B, ns, not significant. (d) PTCH1 genes were detected by RT-qPCR. shGLI1-2 shows the highest silencing efficiency, significantly reducing GLI1 mRNA levels compared to control (con) and nc groups (* p < 0.05). (e) Western blot results confirm reduced GLI1 and PTCH1 protein levels. (f) Morphology of stably transfected cell lines TU212-shPTCH1 and TU212-PTCH1(Scale bar = 100 μm). PTCH1 promotes cell proliferation and regulates cell cycle progression in TU212 cells.

To investigate the effect of silencing GLI1 on GLUL, MAP1B, and PTCH1 genes, three interfering siRNAs (shGLI1−1, shGLI1−2, shGLI1−3) were synthesized, and control con and NC groups were set up. The results showed that shGLI1−2 had the highest silencing efficiency, and its GLI1 mRNA level was significantly lower than that of the Control (Con) and NC groups (Fig. 3a); the different sequences of siRNAs had no significant effect on the GLUL (Fig. 3b) and MAP1B genes (p > 0.05, Fig. 3c), but could significantly reduce the expression of the PTCH1 gene (p < 0.05, Fig. 3d), suggesting that GLI1 could affect the PTCH1 expression. Western blot assay results were consistent with the above description (Fig. 3e).

### Overexpression of PTCH1 promotes EMT and metastasis

**Fig. 4:**
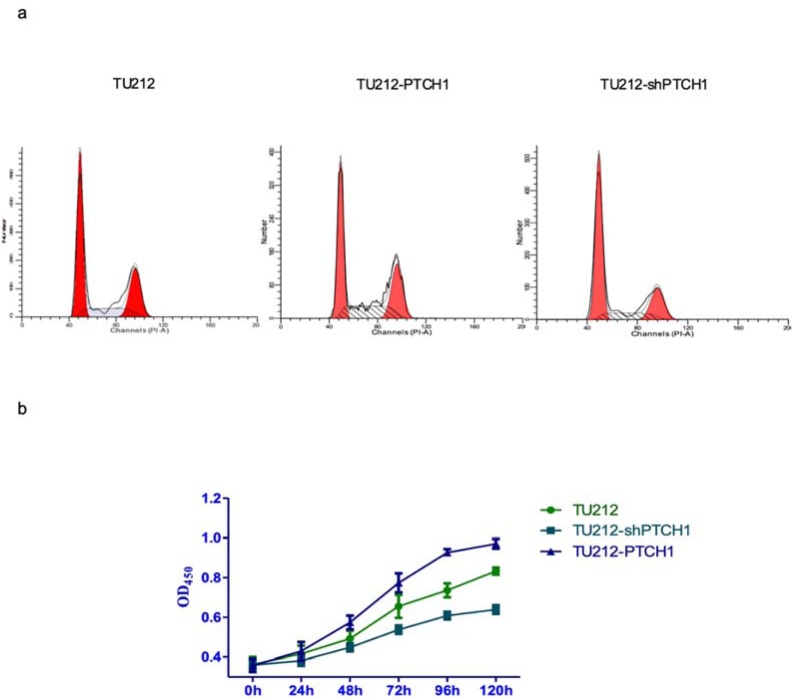
PTCH1 modulates protein expression, cell cycle progression, and proliferation in laryngeal cancer cells. (a) Flow cytometry cell cycle analysis shows PTCH1 overexpression increases G1 phase and decreases S and G2 phases compared to controls. * p < 0.05, while PTCH1 silencing induces S phase arrest. (b) CCK8 assay confirms PTCH1 enhances cell proliferation.

Next, we transfected PTCH1 overexpression vectors into TU212 cells to determine the effects of PTCH1 on EMT, cell migration and invasion (Fig. 3f). The results of flow assay cell cycle showed that silencing PTCH1 induced cell cycle arrest in S phase, while G1 phase was increased and S phase and G2 were decreased in TU212-PTCH1 cells compared with TU212 cells (p < 0.05) (Fig. 4a). Thus, overexpression of PTCH1 could promote cell proliferation. In addition, CCK8 cell proliferation assay showed that PTCH1 promoted cell proliferation (Fig. 4b).

**Fig. 5:**
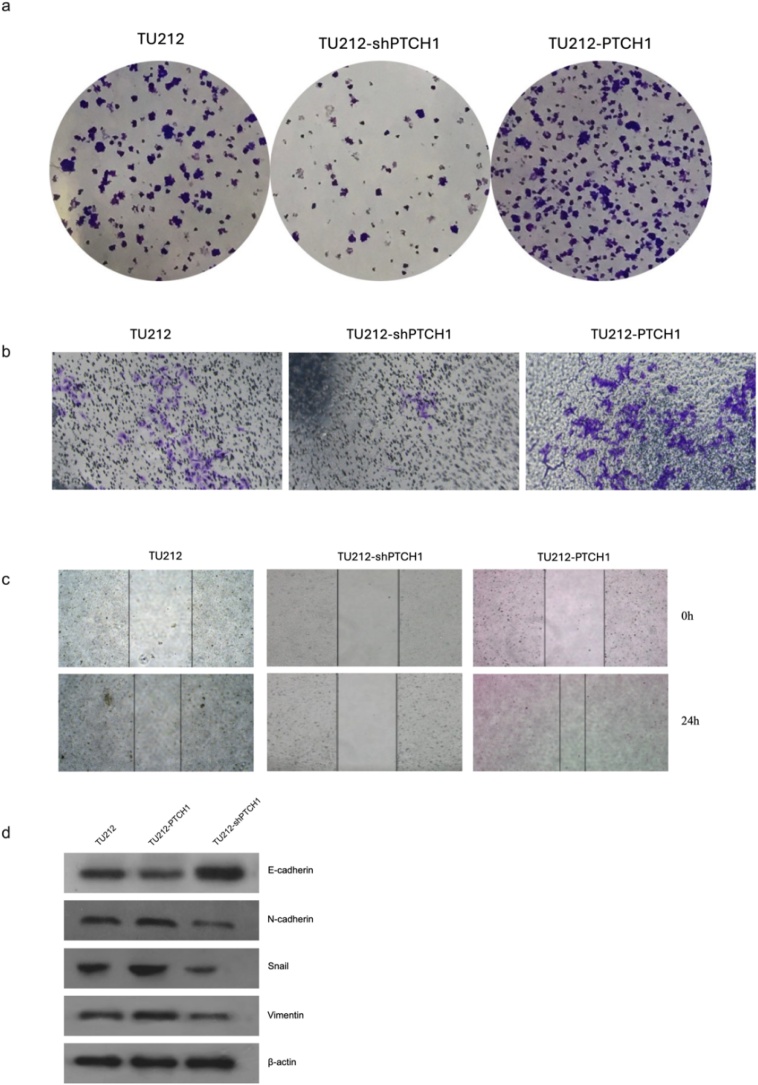
PTCH1 promotes clone formation, invasion, migration, and EMT in TU212 cells. (a) Clone formation assay shows PTCH1 overexpression increases colony formation, while PTCH1 silencing reduces it compared to controls (Scale bar = 200 μm). (b) Invasion assay reveals PTCH1 enhances cell invasion (Scale bar = 500 μm). (c) Cell scratch assay demonstrates PTCH1 overexpression promotes cell migration (Scale bar = 200 μm). (d) Western blot detection of EMT-related protein expression levels of TU212, TU212-shPTCH1 and TU212-PTCH1.

Clone formation assay showed that the number of clone formation in TU212-shPTCH1 cells was significantly lower than that in TU212 cells, whereas the number of clone formation in TU212-PTCH1 cells was significantly higher than that in TU212 (p < 0.05), thus, PTCH1 could promote cell clone formation (Fig. 5a). Invasion assay in TU212 cells showed that compared with empty vector transfection, PTCH1 promoted cell invasion (Fig. 5b). Cell scratch assay showed that PTCH1 overexpression promoted cell migration (Fig. 5c). In addition, we found that in TU212 cells, PTCH1 overexpression led to downregulation of E-cadherin and upregulation of N-cadherin, Snail, and Vimentin, suggesting that PTCH1 may promote EMT (Fig. 5d).

## Discussion

In this study, hsa-mir-1293 was identified as a candidate upstream miRNA of GLI1 through screening miRNA data from The Cancer Genome Atlas (TCGA). Current studies on hsa-mir-1293 have focused on a few tumors, and its 5'-3' sequence, verified using miRBase database data, is UGGGGUGGGUCUGGAGAUUUGUGC. In Lung Adenocarcinoma (LUAD), elevated hsa-mir-1293 expression correlates with advanced pathological stages and poorer overall survival, as it targets PGM5 to regulate LUAD cell malignancy.^26^ Additionally, its peripheral blood expression may serve as a prognostic marker for LUAD patients.^27^ In Non-Small-Cell Lung Cancer (NSCLC), hsa-mir-1293 is upregulated in cisplatin-resistant cell lines, promoting chemoresistance and inhibiting apoptosis by downregulating RUNX3 and activating the PI3K-AKT pathway.^28^ In renal cell carcinoma, high hsa-mir-1293 expression enhances cell viability, migration, and invasion by negatively regulating HAO2,^29^ and it also inhibits DNA repair genes (Apex1, Rpa1, Pold4) and tumor growth in vivo.^30^ Herein, we experimentally confirmed that hsa-mir-1293 regulates laryngeal cancer development via GLI1. Notably, the clinical relevance of the hsa-mir-1293/GLI1/PTCH1 axis is highlighted by its association with the aggressive C2 molecular subtype, which we previously identified in TCGA and GEO cohorts. Given that the C2 subtype exhibits significantly higher malignant potential and poorer prognosis than the C1 subtype, downregulation of hsa-mir-1293, the most significantly altered miRNA in this subtype, provides a molecular explanation for the increased cell proliferation and EMT observed in advanced laryngeal cancer.^4^

When screening for GLI1 downstream target genes in laryngeal cancer cells, PTCH1 was screened by GenCLiP3.0 as target gene that might be recognized and bound by GLI1. PTCH1 serves as the receptor for the Hedgehog (Hh) signaling pathway, with PTCH2 being its mammalian homolog.^31^ In this pathway, Hh proteins are released via exosomes and bind to the PTCH receptor. In the absence of Hh ligands, PTCH inhibits the activity of the Hedgehog signaling pathway.^19^ However, when Hh ligands bind to PTCH1, they relieve PTCH-mediated repression, activating the pathway.^18^ This activation removes the inhibition on Smo, leading to the initiation of the GLI1 transcription factor. The precise mechanism by which PTCH1 regulates Smo remains unclear. Denef et al.^32^ found that Hh binding causes PTCH1 internalization from the cell surface, promoting Smo phosphorylation, stabilization, and aggregation. RNA interference-mediated removal of PTCH1 produces similar effects. The proposed mechanism involves a physical interaction between Smo and PTCH1, forming membrane-associated receptor complexes, though this has not been confirmed in in vivo experiments.^33^

PTCH1, PTCH2 and GLI1 are the 3 main target genes of the Hedgehog signaling pathway, and their activation leads to elevated levels of the corresponding mRNAs and proteins regulated by them, resulting in a cascade response.^34^ Elevated expression of PTCH1, PTCH2 and GLI1 genes is a reliable indicator of activation of the Hedgehog signaling pathway, and GLI1 can provide positive feedback and PTCH1 provide negative feedback to form positive and negative feedback regulation of the Hedgehog signaling pathway.^20^

In this study, silencing of GLI1 significantly decreased the expression of PTCH1 mRNA and protein, so it can be assumed that PTCH1 is a target gene of GLI1 in laryngeal cancer. Shahi et al.^22^ found that transfection of GLI1-targeted siRNAs to silence GLI1 expression in both medulloblastoma and astrocytic cell lines resulted in upregulation of all target genes in medulloblastoma cell lines, whereas only PTCH1 was upregulated in astrocytic tumor. In addition, in tumors such as rhabdomyosarcoma,^23^ chronic granulocytic leukemia,^24^ and multiple myeloma,^21^ PTCH1 is a target gene of GLI1. tGLI1 as a hermaphrodite of GLI1 also promotes retinoblastoma invasion and metastasis by regulating PTCH1.^35^ GLI1 can form a negative feedback regulatory loop of the Hedgehog signaling pathway by regulating PTCH1 transcription.^36^ The in vivo experiments in this study demonstrated that in laryngeal cancer cells PTCH1 can promote cell proliferation, clone formation, cell invasion, and carcinogenicity, thus promoting laryngeal carcinogen.

EMT was first discovered when researchers thought it was just a phenotype switch from epithelial to mesenchymal cells, but a large body of evidence suggests that it plays a key role in stemness, metabolic reprogramming, immune evasion, and therapeutic resistance in cancer cells.^37^ The hallmark of EMT is the loss of expression of epithelial markers (e.g., E-cadherin) and the increased expression of melancholy markers (e.g., N-cadherin and Vimentin) with an invasive phenotype.^38^ Epithelial cells exhibit apical-basal polarity, adhere to a basal lamina, and form tight intercellular junctions, in contrast to mesenchymal cells, which assume a scattered, fibroblastic morphology, lack apical-basal polarity, and are highly motile.^39^ In the present study, overexpression of PTCH1 in laryngeal carcinoma cells reveals the presence of the EMT-associated snail, N adherence and Vimentin protein expression was up-regulated, while E-cadherin protein expression was down-regulated, suggesting that PTCH1 may promote EMT in laryngeal cancer cells, and the specific mechanism needs to be further investigated.

Despite the findings of this study, several limitations exist. Our research was mainly based on the TU212 laryngeal cancer cell line without in vivo validation, which may restrict the generalizability of our conclusions. Nevertheless, the significant correlation between hsa-miR-1293 and GLI1 in TCGA and GEO cohorts supports the clinical significance of our model. Further studies using multiple cell lines, in vivo models, and large clinical samples are needed to verify the therapeutic value of the miR-1293/GLI1/PTCH1 axis.

## Conclusion

In conclusion, we screened for differentially expressed miRNAs by screening miRNA data from TCGA, among which hsa-mir-1293 was most significantly downregulated, and hsa-mir-1293 is a candidate upstream miRNA for GLI1. we also emphasized that hsa-mir-1293 inhibits laryngeal cancer cell proliferation, migration, invasion, and EMT. In addition, GLI1 regulates the target gene PTCH1 thereby affecting the ability of laryngeal cancer cells to proliferate, form clones, and invade cells. The results of this study are important for our understanding of the molecular characteristics of laryngeal cancer and suggest that GLI1 and PTCH1 are potential therapeutic targets for laryngeal cancer.

## ORCID ID

Zhi Chen: 0000-0002-4032-5120

Jun Wan: 0009-0003-5467-5548

Yongchuan She: 0009-0003-3904-1985

Yi Tang: 0009-0000-3958-6465

Dan Yang: 0009-0000-5966-2234

Hong Guo: 0000-0001-8366-6470

Zhongxi Huang: 0000-0003-2389-3166

## Authors' contributions

Yibo Zhou and Yu Ren jointly contributed to the conceptualization and methodology design of the study, and drafted the initial manuscript. Zhi Chen performed data curation and formal analysis, while author Jun Wan was responsible for experimental investigation and validation. The software implementation and visualization were conducted by author Yongchuan She, and author Yi Tang provided essential resources and managed project administration. Dan Yang contributed to critical revision of the manuscript. Hong Guo oversaw the research supervision. Zhongxi Huang as the corresponding author, led the overall supervision, secured funding, and finalized the manuscript editing.

## Fundings

This work was supported by Hunan Provincial Natural Science Foundation Project (2023JJ30068), Changsha Municipal Traditional Chinese Medicine Research Project (SB2024-099), Guided Science and Technology Program Project of Changsha Municipal Science and Technology Bureau (kzd2501082) and Changsha Natural Science Foundation Project (kq2208490, kq2403159).

## Data availability statement

The original contributions presented in the study are included in the Article/Supplementary Material. Further inquiries can be directed to the corresponding author

## Declaration of competing interest

The authors declare that they have no known competing financial interests or personal relationships that could have appeared to influence the work reported in this paper.
