# Supplementary material for: Hsa-mir-1293/GLI1/PTCH1 axis is involved in proliferation, migration, and EMT of laryngeal cancer
Source: Braz J Otorhinolaryngol. 2026 Jul 22;92(5):101860. doi: 10.1016/j.bjorl.2026.101860 (PMC13425815; doi:10.1016/j.bjorl.2026.101860)

BJORL-D-25-00375_Supplementary Material

**Supplementary Table 1** Antibodies used in this study.

| **Target Protein** | **Manufacturer** | **Catalog Number** | **Application** |
| --- | --- | --- | --- |
| GLI1 | Abcam | Ab134906 | WB (1:500) |
| E-cadherin | Abcam | Ab40772 | WB (1:2000) |
| N-cadherin | Abcam | Ab245117 | WB (1:2000) |
| Snail | Abcam | Ab216347 | WB (1:1000) |
| GLUL | Abcam | Ab176562 | WB (1:2000) |
| MAP1B | Abcam | Ab154333 | WB (1:1000) |
| PTCH1 | Abcam | Ab53715 | WB (1:1000) |
| Vimentin | Abcam | Ab92547 | WB (1:500) |
| Hrp-Goat Anti-Rabbit IgG | Jackson | 111-035-003 | WB (1:10000) |
| Hrp-Goat Anti-Mouse IgG | Jackson | 115-035-003 | WB (1:40000) |

**Supplementary Figure S1** Negative correlation between hsa-miR-1293 and GLI1 expression levels. (A) The expression of hsa-mir-1293 in TU212 cells compared to control cells. (B) RT-qPCR analysis of hsa-mir-1293 expression levels. (C) RT-qPCR shows that hsa-miR-1293 negatively regulates GLI1 expression (****p < 0.01).


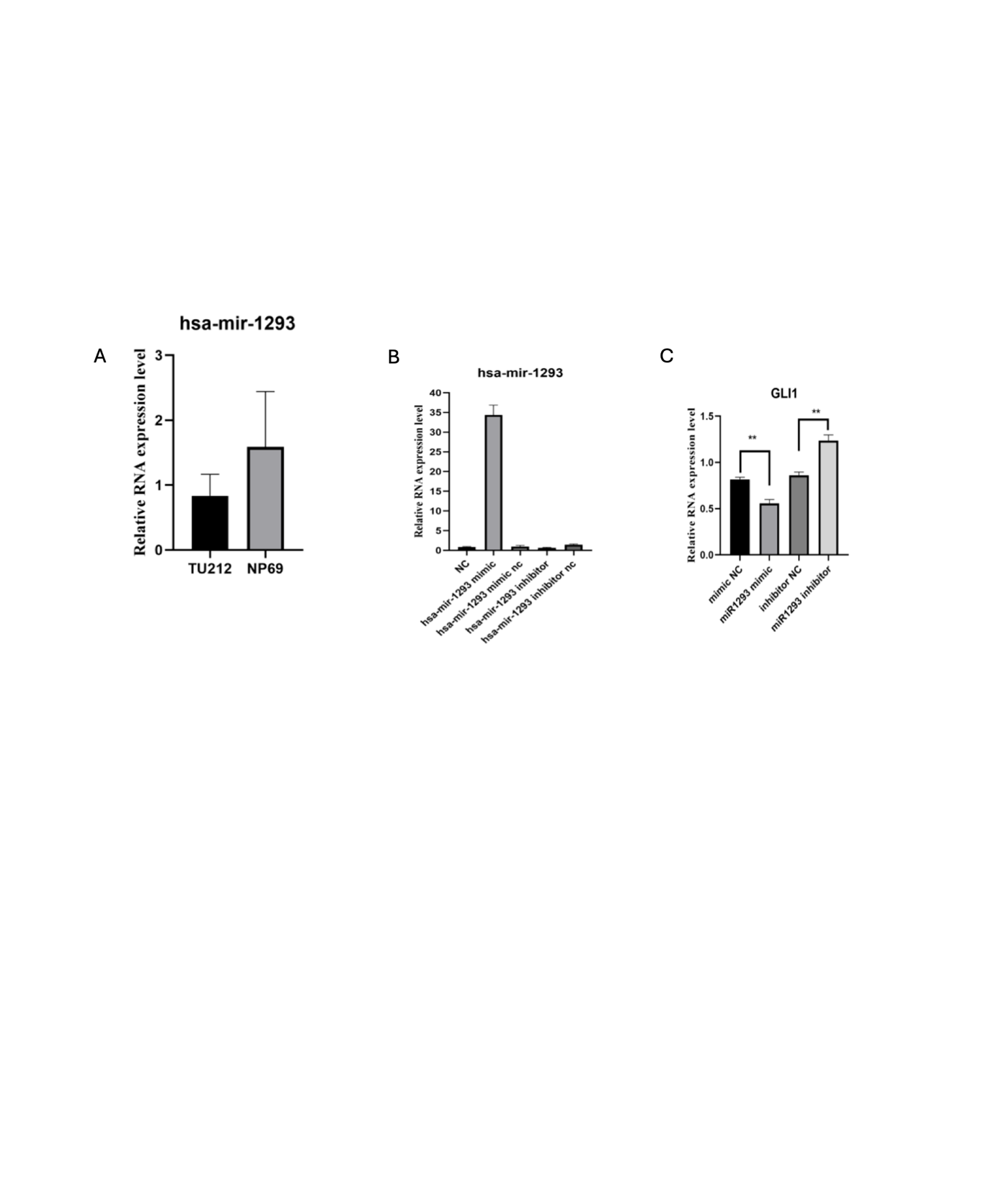


**Supplementary Figure S2** Effects of hsa-miR-1293 on cell cycle distribution in TU212 cells. (A‒B) Flow cytometry reveals that hsa-miR-1293 overexpression increases the proportion of cells in the G1 phase, while its inhibition decreases it (*p < 0.05). (C) mRNA expression levels of TU212, TU212-shPTCH1 and TU212-PTCH1 detected by RT-qPCR. (D) The protein expression levels of TU212, TU212-shPTCH1 and TU212-PTCH1 were detected by Western blot. (E) Gray value analysis of PTCH1 protein expression in modified TU212 cells.

**Supplementary Figure S3** hsa-miR-1293 suppresses malignant behaviors in laryngeal cancer TU212 cells. (A) Quantification of colony numbers. miR-1293 overexpression significantly reduced colony formation (* p < 0.05 vs. NC). (B) Statistical analysis showing miR-1293 inhibition enhanced invasion (* p < 0.05 vs. NC), while mimic group showed no significant difference (ns, p > 0.05). (C) Quantified migration rate demonstrating miR-1293 mimic decreased wound closure (* p < 0.05 vs. NC).


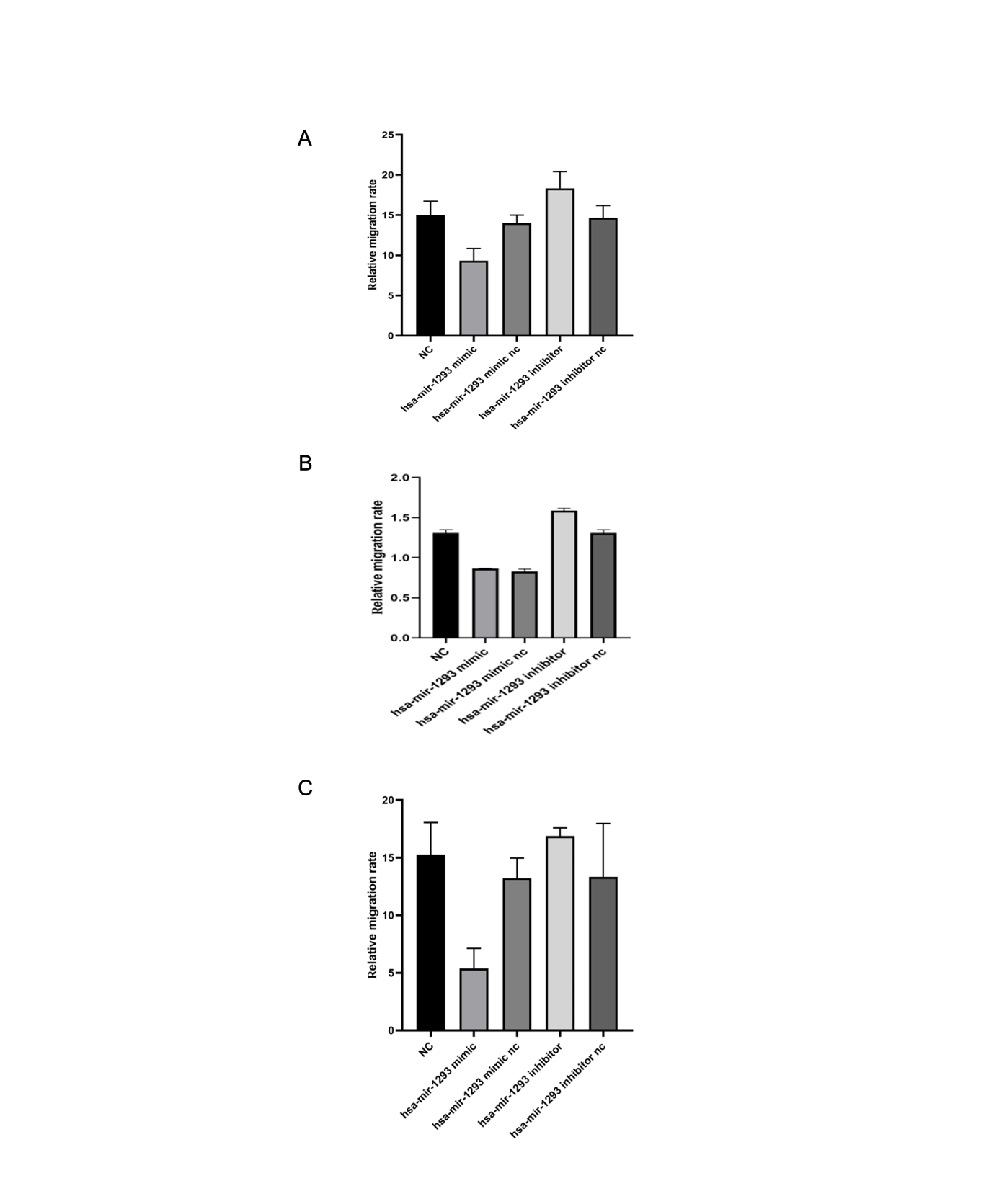


**Supplementary Figure S4** PTCH1 promotes clone formation, invasion, migration, and EMT in TU212 cells. (A) Quantification of colony numbers. Data are mean ± SD (n = 3 independent experiments). *p < 0.05 vs. control group. (B) Statistical analysis of invaded cell counts per field. (C) Quantified migrated area (mm^2^). (D) Densitometric analysis of protein expression levels normalized to GAPDH.

**Supplementary Figure S5** Vector Maps of key plasmids used in this study. (a) GLI1 dual-luciferase reporter plasmid. (b) GLI1 overexpression plasmid. (c‒e) GLI1 shRNA interference plasmid (3 groups).


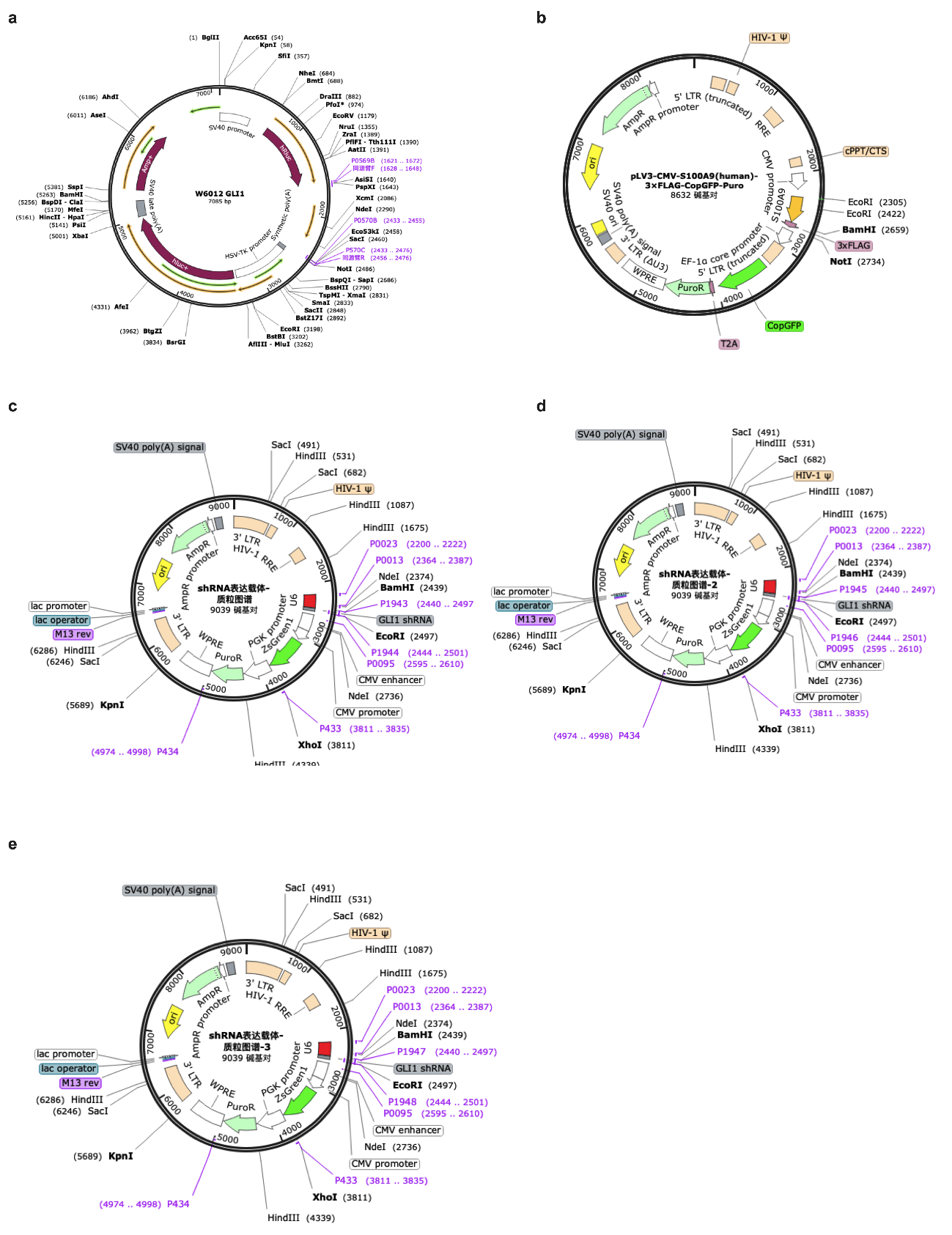

Supplement: Supplementary file 1 [file mmc1.docx]
